# Matrix Polysaccharides and SiaD Diguanylate Cyclase Alter Community Structure and Competitiveness of *Pseudomonas aeruginosa* during Dual-Species Biofilm Development with *Staphylococcus aureus*

**DOI:** 10.1128/mBio.00585-18

**Published:** 2018-11-06

**Authors:** Su Chuen Chew, Joey Kuok Hoong Yam, Artur Matysik, Zi Jing Seng, Janosch Klebensberger, Michael Givskov, Patrick Doyle, Scott A. Rice, Liang Yang, Staffan Kjelleberg

**Affiliations:** a Singapore Centre for Environmental Life Sciences Engineering (SCELSE), Nanyang Technological University, Singapore; b Singapore-MIT Alliance for Research and Technology, Singapore; c University of Stuttgart, Institute of Biochemistry and Technical Biochemistry, Stuttgart, Germany; d Costerton Biofilm Center, Department of International Health, Immunology and Microbiology, University of Copenhagen, Copenhagen, Denmark; e Department of Chemical Engineering, Massachusetts Institute of Technology, Cambridge, Massachusetts, USA; f School of Biological Sciences, Nanyang Technological University, Singapore; g The ithree Institute, The University of Technology Sydney, Sydney, Australia; h Center for Marine Bio-Innovation and School of Biotechnology and Biomolecular Sciences, University of New South Wales, Sydney, Australia; University of Hawaii at Manoa; University of Nottingham; Bar-Ilan University; The Ohio State University

**Keywords:** *Pseudomonas aeruginosa*, SiaD, *Staphylococcus aureus*, biofilms, cyclic di-GMP, exopolysaccharide, microrheology

## Abstract

Mixed-species biofilms display a number of emergent properties, including enhanced antimicrobial tolerance and communal metabolism. These properties may depend on interspecies relationships and the structure of the biofilm. However, the contribution of specific matrix components to emergent properties of mixed-species biofilms remains poorly understood. Using a dual-species biofilm community formed by the opportunistic pathogens Pseudomonas aeruginosa and Staphylococcus aureus, we found that whilst neither Pel nor Psl polysaccharides, produced by P. aeruginosa, affect relative species abundance in mature P. aeruginosa and S. aureus biofilms, Psl production is associated with increased P. aeruginosa abundance and reduced S. aureus aggregation in the early stages of biofilm formation. Our data suggest that the competitive effect of Psl is not associated with its structural role in cross-linking the matrix and adhering to P. aeruginosa cells but is instead mediated through the activation of the diguanylate cyclase SiaD. This regulatory control was also found to be independent of the siderophore pyoverdine and *Pseudomonas* quinolone signal, which have previously been proposed to reduce S. aureus viability by inducing lactic acid fermentation-based growth. In contrast to the effect mediated by Psl, Pel reduced the effective crosslinking of the biofilm matrix and facilitated superdiffusivity in microcolony regions. These changes in matrix cross-linking enhance biofilm surface spreading and expansion of microcolonies in the later stages of biofilm development, improving overall dual-species biofilm growth and increasing biovolume severalfold. Thus, the biofilm matrix and regulators associated with matrix production play essential roles in mixed-species biofilm interactions.

## Abstract (precis)

Bacteria in natural and engineered environments form biofilms that include many different species. Microorganisms rely on a number of different strategies to manage social interactions with other species and to access resources, build biofilm consortia, and optimize growth. For example, Pseudomonas aeruginosa and Staphylococcus aureus are biofilm-forming bacteria that coinfect the lungs of cystic fibrosis patients and diabetic and chronic wounds. P. aeruginosa is known to antagonize S. aureus growth. However, many of the factors responsible for mixed-species interactions and outcomes such as infections are poorly understood. Biofilm bacteria are encased in a self-produced extracellular matrix that facilitates interspecies behavior and biofilm development. In this study, we examined the poorly understood roles of the major matrix biopolymers and their regulators in mixed-species biofilm interactions and development.

## INTRODUCTION

Bacteria exist predominantly as dense, self-organized communities encased in self-produced matrices known as biofilms (1, 2). They exhibit emergent properties that are not found in their single-cell planktonic counterparts, such as altered and enhanced metabolic efficiency (3–5), increased robustness and resistance to antimicrobial attack (6, 7), altered virulence (8, 9), and enhanced horizontal gene transfer (10, 11). These emergent properties contribute to their roles in the Earth’s natural cycling of nitrogen and sulfur and of many metals (12–14) as well as in survival in host organisms, where they can live as commensals or as pathogens (14). While biofilms usually encompass a large diversity of bacterial species that have synergistic, mutualistic, competitive, or antagonistic relationships, the fundamental mechanisms that drive mixed-species biofilm development and the associated emergent properties remain poorly understood.

Pseudomonas aeruginosa and Staphylococcus aureus are opportunistic pathogens found in infections of cystic fibrosis (CF) lungs and in diabetic and chronic wounds (14, 15). Such mixed-species infections are correlated with poor clinical outcomes (16); hence, the two organisms serve as a model dual-species community to represent polymicrobial infections (17, 18). The two bacterial species are known to have an antagonistic relationship, where P. aeruginosa produces heptyl-4-hydroxyquinoline N-oxide (HQNO), a potent inhibitor of respiratory electron transfer and a component of its *Pseudomonas* quinolone signal (PQS) system, to kill S. aureus (19). However, this also selects for S. aureus small-colony variants (SCVs) that have mutations in the electron transport chain and increased resistance to P. aeruginosa killing (20). This has an impact on disease prognosis, as the prevalence of S. aureus SCVs is correlated with a more severe disease state (16). P. aeruginosa also induces the production of the host enzyme sPLA2-IIA to kill S. aureus (21). While these two species serve as a model system for polymicrobial infections, the mechanisms of interaction during dual-species biofilm formation has been less extensively explored.

P. aeruginosa is known to express three polysaccharides, alginate, Pel, and Psl, as the major matrix components (22). P. aeruginosa isolates from the cystic fibrosis (CF) lung environment tend to become mucoid through overexpression of alginate (23). However, only Pel and Psl have been shown to be required for biofilm formation (24, 25). Psl is important for surface attachment (24, 26, 27), autoaggregative phenotypes in batch cultures (28–30), and activation of specific enzymes (diguanylate cyclases [DGCs]) to increase intracellular levels of cyclic-di-GMP, triggering P. aeruginosa to enter the biofilm mode of life (31, 32). Thus, the loss of Psl results in delayed biofilm development and either a delay in or loss of microcolony formation (25, 26, 33). Pel is often associated with the formation of floating biofilms (pellicles) and plays a role in biofilm maturation (24, 26, 33). Pel and Psl have different mechanical properties and resistances to flow that result in differences in biofilm structure and development (33). In mucoid P. aeruginosa*-*S. aureus biofilms, Psl expression led to P. aeruginosa exclusively occupying the upper layer of biofilms, whereas Pel expression appeared to increase colocalization of P. aeruginosa and S. aureus (33).

Recently, it was found that protein A, a cell wall protein of S. aureus, binds to the Psl polysaccharide and type IV pili in P. aeruginosa to inhibit biofilm formation (34). The Psl polysaccharide is also known to affect the community structure and stress resistance, where it confers antibiotic protection to the Escherichia coli-S. aureus biofilm community (35), as well as increased P. aeruginosa abundance and SDS tolerance of three-species biofilms of P. aeruginosa, Pseudomonas protegens, and Klebsiella pneumoniae (36). Thus, the composition of the biofilm matrix represents an important and yet largely underexplored mediator of interspecies interactions and confers emergent properties to the community.

To address how Pel and Psl affect P. aeruginosa competitiveness, biofilm structure, and rheology in mixed-species biofilm communities, we established dual-species biofilms of P. aeruginosa and S. aureus. We explored the importance of the structural role of Psl in the biofilm matrix through analysis of the adhesin CdrA, which physically binds P. aeruginosa cells to the Psl matrix (28), and the regulatory role of Psl in biofilm formation through analysis of the diguanylate cyclases SadC and SiaD, which are activated by Psl to increase c-di-GMP levels (31, 32). In this study, we demonstrate that Psl enables wild-type P. aeruginosa to outcompete S. aureus in early biofilm development and that SiaD is necessary for P. aeruginosa to outcompete S. aureus in a pyoverdine- and PQS-independent manner. In late-stage biofilm development, the production of Pel is required to reduce the effective cross-linking of the matrix to increase the spreading surface coverage of P. aeruginosa in dual-species biofilms.

## RESULTS

### The accumulation of P. aeruginosa in mixed biofilms with S. aureus is facilitated by Psl during early biofilm formation whereas Pel mediates biofilm maturation.

**FIG 1:**
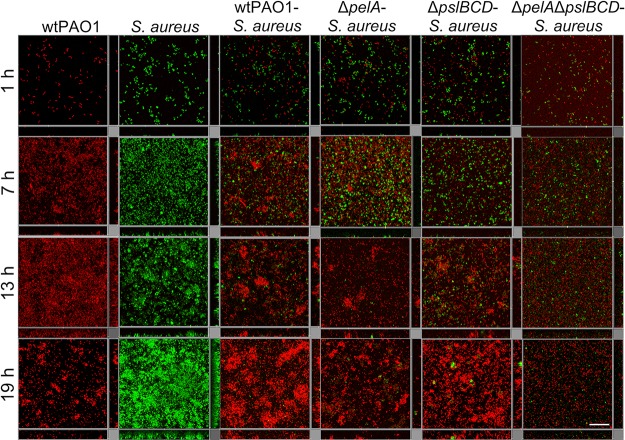
The development of 19-h wild-type P. aeruginosa-S. aureus, P. aeruginosa Δ*pelA*-S. aureus, and P. aeruginosa Δ*pslBCD*-S. aureus dual-species biofilms imaged using confocal imaging every 6 h. The monospecies wild-type P. aeruginosa and S. aureus biofilms are shown for comparison. P. aeruginosa is mCherry tagged (red), and S. aureus is Gfp tagged (green). Images are representative of four biological replicates. The scale bar is 30 μm.

mCherry-tagged, wild-type P. aeruginosa PAO1 or its isogenic Pel and Psl mutants (Table 1) was cocultivated with Gfp-tagged wild-type S. aureus 15981 to examine the impact of the matrix polysaccharides Pel and Psl on the development of P. aeruginosa-S. aureus biofilms (Fig. 1). Wild-type monospecies biofilms, seeded with the same total cell densities as the dual-species biofilms, were also formed for comparison to wild-type dual-species biofilms. The biovolume of each species in the biofilms was calculated using COMSTAT (Table 2). At 1 h, the monospecies wild-type biofilms had total biovolumes of 26,286 ± 4,128 μm^3^ mm^−2^ (P. aeruginosa) and 39,168 ± 2,660 μm^3^ mm^−2^ (S. aureus). This was similar to the results seen with dual-species biofilms with a total biovolume of 28,419 ± 4,586 μm^3^ mm^−2^, where the two species were present in approximately equal amounts. For monospecies wild-type P. aeruginosa, the highest biovolume of 1,548,912 ± 682,644 μm^3^ mm^−2^ was reached at 13 h and decreased to 277,455 ± 292,722 μm^3^ mm^−2^ at 19 h. Monospecies wild-type S. aureus had lower biovolumes (851,130 ± 292,722 μm^3^ mm^−2^) than monospecies wild-type P. aeruginosa at 13 h, but its biovolumes increased to 4,068,969 ± 1,335,431 μm^3^ mm^−2^ by 19 h.

**TABLE 1:** List of bacterial strains^*a*^

Strain	Relevant characteristic(s)	Reference or source
*P. aeruginosa* wtPAO1	Wild-type strain	64
*P. aeruginosa* Δ*pelA*	PAO1 that does not produce the Pel matrix polysaccharide	53
*P. aeruginosa* Δ*pslBCD*	PAO1 that does not express the Psl matrix polysaccharide	53
*P. aeruginosa* Δ*cdrA*	PAO1 lacking the extracellular adhesin CdrA	This study
*P. aeruginosa* Δ*sadC*	PAO1 lacking the DGC SadC	51
*P. aeruginosa* Δ*siaD*	PAO1 lacking the DGC SiaD	51
*P. aeruginosa* Δ*siaD*/pUC18-*siaD*	Carb^r^; ΔSiaD mutant containing the pUC18-*siaD* complementation plasmid	This study
*S. aureus* 15981	Wild-type strain	53

a wtPAO1, wild-type strain PAO1; Carb^r^, carboxylate resistance.

**TABLE 2:** Biovolumes for single- and dual-species biofilms^*a*^

Biofilm	Strain(s)	Biovolume per area (μm^3^ mm^−2^)			
		1 h	7 h	13 h	19 h
Monospecies: *P. aeruginosa*	wtPAO1	26,286 ± 4,128	542,412 ± 260,428	1,548,912 ± 682,644	277,455 ± 59,146
Monospecies: *S. aureus*	*S. aureus* 15981	39,168 ± 2,660	495,781 ± 190,117	851,130 ± 292,722	4,068,969 ± 1,335,431
Dual species: *P. aeruginosa*- *S. aureus*	wtPAO1	14,388 ± 2,168	73,488 ± 21,746	837,343 ± 104,916	792,562 ± 10,272
	*S. aureus* 15981	14,031 ± 2,418	35,015 ± 18,203	12,785 ± 8,189	10,521 ± 3,878
Dual species: mutant Δ*pelA*-*S. aureus*	Mutant Δ*pelA*	14,285 ± 2,693	82,682 ± 32,691	220,077 ± 52,939*	267,374 ± 114,530*
	*S. aureus* 15981	13,045 ± 1,993	128,861 ± 62,036	6,927 ± 2,785	14,982 ± 4,887
Dual species: mutant Δ*pslBCD*-*S. aureus*	Mutant Δ*pslBCD*	14,722 ± 799	14,992 ± 4,185	178,387 ± 48,490*	1,419,718 ± 432,200
	*S. aureus* 15981	17,662 ± 1,729	62,035 ± 15,636	67,605 ± 31,815	20,726 ± 5,301
Dual species: mutant Δ*pelA* Δ*pslBCD*-*S. aureus*	Mutant Δ*pelA* Δ*pslBCD*	7,611 ± 513*	23,988 ± 2,379	21,310 ± 4,559*	50,401 ± 21,795*
	*S. aureus* 15981	7,691 ± 2,315	17,752 ± 5,103	4,559 ± 1,818	1,868 ± 701

a Data represent results from four biological replicates with each replicate composed of three confocal images of the biofilm in different areas on average. Asterisks (*) indicate a significant difference from the wild-type *P. aeruginosa-S. aureus* biofilms (*P *<* *0.05) (unpaired *t* test with Welch’s correction). Error data represent SEM.

For dual-species wild-type P. aeruginosa-S. aureus, the highest total biovolume was 850,128 ± 113,105 μm^3^ mm^−2^ at 13 h, with S. aureus comprising only 1.5% or 12,785 μm^3^ mm^−2^ of the biovolume. These biovolume levels were unchanged at 19 h, which contrasts with the monospecies biofilms of P. aeruginosa. This would suggest that P. aeruginosa biofilms persist longer in the presence of S. aureus. S. aureus made up a minor portion of biovolumes in the dual-species biofilm and reached a peak biovolume of 35,015 ± 18,203 μm^3^ mm^−2^ at 7 h, in contrast to the biovolume seen when it was grown as a monospecies biofilm (495,781 ± 190,117 μm^3^ mm^−2^). This indicates that P. aeruginosa inhibited S. aureus growth during early biofilm development.

**FIG 2:**
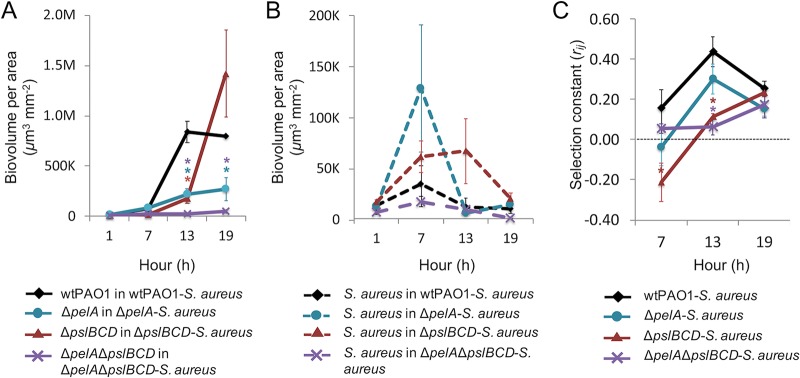
The development of 19-h biofilms formed by wild-type and matrix mutants of P. aeruginosa cocultured with S. aureus. Error bars represent standard errors of the means (SEM) (*n* ≥ 4). *, *P < *0.05, α = 0.05 (unpaired two-sided *t* test with Welch’s correction). (A) Biovolumes per area of P. aeruginosa (solid lines) in the dual-species biofilms every 6 h. (B) Biovolumes per area of S. aureus (dashed lines) in the dual-species biofilms every 6 h. Note that the *y* axis scales for panels A and B are different. (C) Fitness of P. aeruginosa relative to S. aureus, where a selection constant of *r_ij_* = 0 means that P. aeruginosa and S. aureus are equally competitive and a *r_ij_* value of >0 means P. aeruginosa is more competitive than S. aureus.

For all dual-species P. aeruginosa-S. aureus biofilms, the biovolume of P. aeruginosa was roughly equal to the biovolume of S. aureus in the initial phase of biofilm formation (i.e., after 1 h of inoculation). The average biovolume for each species ranged from 13,045 to 17,662 μm^3^ mm^−2^, with the exception of mutant Δ*pelA* Δ*pslBCD*-S. aureus biofilms, where each species displayed approximately half those biovolumes (Fig. 2A) (Table 2).

During dual-species biofilm development, the average biovolume for both wild-type P. aeruginosa and the Δ*pelA* mutant increased by approximately 5-fold during the first 7 h. The average biovolume of wild-type P. aeruginosa increased by 10-fold at 13 h and remained relatively constant at 792,562 ± 10,272 μm^3^ mm^−2^ at 19 h. The Δ*pelA* mutant increased in biovolume by 3-fold to 4-fold between 7 and 13 h and remained constant at 267,374 ± 114,530 μm^3^ mm^−2^ from 13 to 19 h. For the Δ*pslBCD* mutant, the average biovolume was unchanged at 7 h but was found to have increased by approximately 10-fold at 13 h and a further 10-fold by 19 h to achieve a final biovolume of 1,419,718 ± 432,200 μm^3^ mm^−2^. For the Δ*pelA* Δ*pslBCD* mutant, the average biovolume increased in 3-fold increments at each 6-h time point to a final average biovolume of 50,401 ± 21,795 μm^3^ mm^−2^ at 19 h. Thus, the loss of Pel in the dual-species biofilms was associated with an overall reduction in total biovolume at 13 and 19 h (Fig. 2A).

The average biovolume of S. aureus for all biofilms increased initially but remained low throughout biofilm development compared to P. aeruginosa and by 19 h had returned to levels similar to or lower than those observed at the start of biofilm formation (Table 2) (Fig. 2B). There were no significant differences in the S. aureus biovolumes of the dual-species biofilms with different combinations of Pel and Psl expression.

The selection constant rates (*r_ij_*), representing the ratios of P. aeruginosa over S. aureus over time, were derived from the biovolume data to determine the competitiveness of the P. aeruginosa wild-type and polysaccharide mutants against S. aureus (Table 3, columns 2 to 4). Only the Δ*pslBCD* mutant was less competitive than S. aureus after 7 h of biofilm formation (Table 3, columns 5 and 6) (Fig. 2C), and the *r_ij_* value was significantly different from that determined for the wild-type (*P = *0.04, α = 0.05, *n* = 4). At 13 h, the Δ*pslBCD* mutant was more competitive than S. aureus but the competitiveness was still lower than and significantly different from that seen with the wild-type (*P = *0.02, α = 0.05, *n* = 4). The Δ*pelA* Δ*pslBCD* mutant was significantly less competitive than the wild-type strain against S. aureus at 13 h (*P = *0.01, α = 0.05, *n* = 4) (Table 3, columns 2 to 4) (Fig. 2C). By 19 h, both the Δ*pslBCD* and Δ*pelA* Δ*pslBCD* mutants were as competitive as the wild-type strain against S. aureus.

**TABLE 3:** Competitiveness of *P. aeruginosa* relative to *S. aureus*

Biofilm	Selection rate constant, *r_ij_*^*a*^			Correlation of fitness curves^*b*^	
	7 h	13 h	19 h	Biofilm	*r_*XY*_*
wtPAO1-*S. aureus*	0.16 ± 0.09	0.43 ± 0.07	0.25 ± 0.04	wtPAO1-*S. aureus* with Δ*pelA*-*S. aureus*	0.97
Mutant Δ*pelA*-*S. aureus*	−0.04 ± 0.20	0.30 ± 0.06	0.15 ± 0.04	wtPAO1-*S. aureus* with Δ*pslBCD*-*S. aureus*	0.58
Mutant Δ*pslBCD*-*S. aureus*	−0.21 ± 0.09*	0.12 ± 0.01*	0.23 ± 0.02	Δ*pelA*-*S. aureus* with Δ*pslBCD*-*S. aureus*	0.76
Mutant Δ*pelA* Δ*pslBCD*-*S. aureus*	0.06 ± 0.02	0.07 ± 0.04*	0.18 ± 0.07	wtPAO1-*S. aureus* with Δ*pelA* Δ*pslBCD*-*S. aureus*	-0.11
Mutant Δ*siaD*-*S. aureus*	0.11 ± 0.06	0.07 ± 0.04*	0.01 ± 0.01*	Δ*pelA*-*S. aureus* with Δ*pelA* Δ*pslBCD*-*S. aureus*	0.13
Mutant Δ*siaD*(*siaD*)-*S. aureus*	0.23 ± 0.05	0.19 ± 0.04	0.18 ± 0.05	Δ*pslBCD*-*S. aureus* with Δ*pelA* Δ*pslBCD*-*S. aureus*	0.75

a Data represent selection rate constants during 19 h of biofilm development determined from four biological replicates, with each replicate derived from an average of three confocal images. Asterisks (*) indicate a significant difference from the wild-type *P. aeruginosa-S. aureus* biofilms (*P *<* *0.05) (unpaired *t* test with Welch’s correction). Error data represent SEM.

b Correlation of fitness curves is given by the Pearson correlation coefficient (*r_XY_*).

Although the Δ*pelA* mutant was less competitive than the wild-type strain against S. aureus throughout biofilm formation, the differences were not statistically significant (Table 3, columns 2 to 4) (Fig. 2C). Based on the *r_ij_* values, changes in the competitiveness of the Δ*pelA* mutant and wild-type P. aeruginosa were also similar for the dual-species biofilms, with *r_ij_* reaching a peak at 13 h and the *r_ij_* over time (fitness curves), displaying a strong positive correlation of *r_XY_* = 0.97 (Table 3, columns 5 and 6) (Fig. 2C). Thus, Psl contributes to the competitive fitness of the wild-type strain only during the early stages of P. aeruginosa-S. aureus biofilm development.

### S. aureus and Pel production are associated with an increase in surface coverage and in the microcolony size of P. aeruginosa in dual-species biofilms.

**FIG 3:**
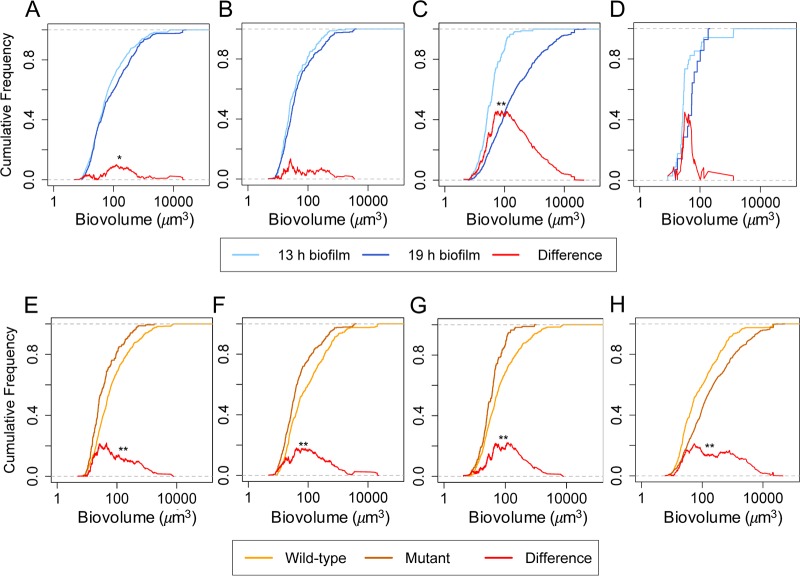
Comparison of microcolony sizes in dual-species biofilms using the two-sample, two-sided Kolmogorov-Smirnov test. The red curve shows the difference between the two distributions (*, *P < *0.05, **, *P < *0.01, ***, *P < *0.001; α = 0.05). (Top panel) Changes in size distribution as biofilms progresses from 13 to 19 h. (A to D) P. aeruginosa microcolonies in (A) wild-type P. aeruginosa-S. aureus, (B) mutant Δ*pelA*-S. aureus, and (C) mutant Δ*pslBCD*-S. aureus biofilms and S. aureus microcolonies in (D) mutant Δ*pslBCD*-S. aureus biofilms. (Bottom panel) Differences between P. aeruginosa microcolony size distributions formed by wild-type and matrix mutants of P. aeruginosa cocultured with S. aureus. (E and F) Wild-type P. aeruginosa-S. aureus compared to mutant Δ*pelA*-S. aureus at (E) 13 h and (F) 19 h. (G and H) Wild-type P. aeruginosa-S. aureus compared to mutant Δ*pslBCD*-S. aureus at (G) 13 h and (H) 19 h.

To explore how Pel and Psl affected the structure of dual-species biofilms (Fig. 1), the average surface coverage, the number of microcolonies, and the microcolony sizes of 13-h and 19-h biofilms were calculated (Table 4). Monospecies wild-type biofilms were also investigated for comparison to the dual-species, wild-type biofilms (Table 4). Microcolonies included small and large cell clusters, which ranged in size over various orders of magnitude (Fig. 3).

**TABLE 4:** Surface coverage, microcolony size, and number of microcolonies in single- and mixed-species biofilms^*a*^

Biofilm	Strain(s)	Avg surface coverage (%)		Avg no. of microcolony per area (mm^−2^)		Avg microcolony biovolume (μm^3^)	
		13 h	19 h	13 h	19 h	13 h	19 h
Monospecies: *P. aeruginosa*	wtPAO1	14 ± 4	10 ± 2	1,757 ± 386	1,974 ± 360	109 ± 46	58 ± 23
							
Monospecies: *S. aureus* 15981	*S. aureus* 15981	20 ± 5	40 ± 10	2,520 ± 552	3,325 ± 507	1,346 ± 3,035	7,356 ± 2,279
							
Dual species: *P. aeruginosa*- *S. aureus*	wtPAO1	19 ± 3	22 ± 0	5,265 ± 489	3,716 ± 462	108 ± 7	499 ± 91
	*S. aureus* 15981	0 ± 2	0 ± 0				
							
Dual species: mutant Δ*pelA*-*S. aureus*	Mutant Δ*pelA*	8 ± 1	9 ± 3	1,025 ± 302	2,581 ± 1,117	67 ± 5	111 ± 19
	*S. aureus* 15981	0 ± 0	1 ± 0				
							
Dual species: mutant Δ*pslBCD*-*S. aureus*	Mutant Δ*pslBCD*	7 ± 3	33 ± 14	1,043 ± 756	4,661 ± 2,407	50 ± 4	1,400 ± 358
	*S. aureus* 15981	2 ± 1	1 ± 0	207 ± 138	85 ± 85	65 ± 16	65 ± 15
							
Dual species: mutant Δ*pelA* Δ*pslBCD*-*S. aureus*	Mutant Δ*pelA* Δ*pslBCD*	3 ± 1	5 ± 2				
	*S. aureus* 15981	1 ± 0	0 ± 0				

a Values are derived from three biological replicates, with each replicate derived from an average of three confocal images. Error data represent SEM.

Wild-type P. aeruginosa displayed the highest surface coverage at 13 h compared to the matrix mutants in the dual-species biofilms. Wild-type P. aeruginosa had a surface coverage of 19% ± 3%, while the Δ*pelA* and Δ*pslBCD* mutants had similar surface coverages at 8% ± 1% and 7% ± 3%, respectively, and the Δ*pelA* Δ*pslBCD* mutant displayed a surface coverage of 3% ± 1% (Table 4). At 19 h, biofilms of wild-type P. aeruginosa and mutants Δ*pelA* and Δ*pelA* Δ*pslBCD* were similar to the 13-h biofilms at 22% ± 0%, 9% ± 3%, and 5% ± 2% surface coverage, respectively, whereas the Δ*pslBCD* mutant surface coverage increased to 33% ± 14% (Table 4). The surface coverage of S. aureus in the dual-species biofilms at 13 and 19 h ranged from 0% to 2% irrespective of whether wild-type or mutant P. aeruginosa was included (Table 4). P. aeruginosa had more surface coverage in dual-species biofilms than in monospecies biofilms at both 13 and 19 h. However, S. aureus had much less surface coverage at 13 and 19 h in cocultures with P. aeruginosa (Table 4).

Wild-type P. aeruginosa formed the greatest number of microcolonies compared to the mutants in the dual-species biofilms. The average microcolony size was 108 ± 7 μm^3^ at 13 h and increased to 499 ± 91 μm^3^ by 19 h (Table 4) (Fig. 3A). Wild-type P. aeruginosa also formed more microcolonies as a dual-species biofilm than as a monospecies biofilm. The sizes of the microcolonies formed by P. aeruginosa in dual-species biofilms and monospecies biofilms were similar at 13 h, but the microcolonies in the dual-species biofilms were about 10 times larger than in the monospecies biofilms by 19 h.

The Δ*pelA* mutant formed approximately 5-fold-fewer microcolonies than the wild-type strain, and the average microcolony size was 67 ± 5 μm^3^ at 13 h. By 19 h, the number of microcolonies had not increased, but the average microcolony size increased to 111 ± 19 μm^3^ (Table 4). The microcolony size distributions were not significantly different as the biofilm progressed from 13 to 19 h (Fig. 3B). The Δ*pelA* microcolony size was significantly smaller than the wild-type microcolony size at both 13 h (Fig. 3E) and 19 h (Fig. 3F).

The Δ*pslBCD* mutant also formed approximately 5-fold-fewer microcolonies than the the wild-type strain by 13 h, and the average microcolony size was 50 ± 4 μm^3^. By 19 h, the number of microcolonies had increased by ∼5-fold and the average microcolony size had increased to 1,400 ± 358 μm^3^ (Table 4). The microcolony size distribution at 19 h was larger than and significantly different from that seen of 13 h of biofilm development, with the difference between the two distributions being as large as 46% (Fig. 3C). The Δ*pslBCD* microcolony size distribution was smaller than that of the wild-type strain at 13 h (Fig. 3G) but was larger at 19 h (Fig. 3H).

The Δ*pelA* Δ*pslBCD* mutant did not form microcolonies in the dual-species biofilms. S. aureus also did not form microcolonies in any of the dual-species biofilms except when cultivated with the Δ*pslBCD* strain, with average microcolony sizes of 65 ± 16 μm^3^ at 13 h and 65 ± 15 μm^3^ by 19 h (Fig. 3D). Thus, expression of Psl was required for microcolony formation of P. aeruginosa and hindered S. aureus biofilm formation. Expression of Pel was required for expanding surface coverage and microcolonies.

### Pel and Psl have opposing rheological roles in the matrix of the microcolonies.

Biofilm rheological properties conferred by Pel and Psl affect biofilm structure and spreading at different stages in monospecies P. aeruginosa biofilms under flow conditions (33). Specifically, Psl cross-links the biofilm to increase microcolony formation and reduce spreading at the early stages, while Pel loosens the biofilm to increase spreading at the later stages of biofilm development (33). Thus, we investigated the rheological properties of 19-h dual-species biofilms using particle-tracking microrheology (37, 38) to determine if the mechanical roles of Pel and Psl in monospecies flow cell biofilms were maintained in P. aeruginosa-S. aureus static biofilms. If so, this could explain the increased surface coverage and microcolony sizes observed in P. aeruginosa-S. aureus biofilms expressing Pel.

**FIG 4:**
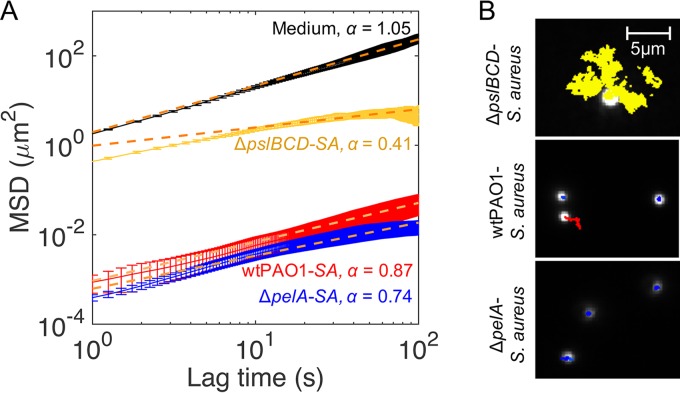
Microrheological measurements of wild-type and matrix mutants of P. aeruginosa cocultured with S. aureus. (A) MSD curves for wild-type P. aeruginosa-S. aureus, mutant Δ*pelA*-S. aureus, and mutant Δ*pslBCD*-S. aureus microcolonies. The MSD curve for TSB medium is shown for comparison. *SA*, S. aureus. The orange dotted lines indicate the line of best fit to the experimentally determined MSD using a power law function for the estimation of α. (B) Representative particle trajectories in wild-type P. aeruginosa-S. aureus, mutant Δ*pelA*-S. aureus, and mutant Δ*pslBCD*-S. aureus microcolonies. The red trajectory in the middle panel indicates a particle undergoing directed motion and superdiffusion, whereas the blue and yellow trajectories indicate subdiffusion. Error bars represent SEM.

The mean squared displacement (MSD) of particles embedded within microcolonies of 19-h biofilms was directly proportional to their creep compliance (*J*) and effective cross-linking (39, 40). The changes in effective cross-linking could have been due to differences in polymer chain length and concentration and degree of polymer entanglement and to interactions between different polymers. The interactions between polymers can be chemical (operating through covalent bonds), physical (operating through noncovalent interactions), or topological, depending on the polymeric entanglements. The MSDs were plotted as a function of lag time (elapsed time; 1 s ≤ *t *≤* *100 s) to give the MSD curves from which the power law exponent (α) can be derived. When α = 1, the particle is considered to be undergoing normal diffusion; when α < 1, this is termed subdiffusion; and when α > 1, they are considered to be undergoing superdiffusion (41, 42). The diffusive regime also informs one of the rheological environment in which the particle is embedded. For example, when α = 0, the substance is purely elastic; when 0 < α < 1, the substance is viscoelastic; and when α = 1, the substance is purely viscous (37, 38). The undifferentiated layers in wild-type P. aeruginosa-S. aureus and mutant Δ*pelA*-S. aureus that trapped the particles were too thin for investigation without an attachment to or a capillary effect from the substratum and hence were not investigated. The rheological parameters of the sterile TSB medium (17 g liter^−1^ casein peptone, 2.5 g liter^−1^ K_2_HPO_4_, 2.5 g liter^−1^ glucose, 5 g liter^−1^ NaCl, 3 g liter^−1^ soya peptone) were characteristic of a viscous Newtonian fluid at α = 1.04 and *J *=* *11,500 ± 789 Pa^−1^ at *t *=* *10^1^ s (Fig. 4A) (Table 5). In comparison, the wild-type P. aeruginosa-S. aureus microcolonies displayed creep compliance of *J *=* *3 ± 2 Pa^−1^ at *t *=* *10^1^ s and a power law exponent α value of 0.87 (Fig. 4A). In addition, several particles were observed to undergo superdiffusion, which was also defined by their directional movement, with a net displacement of 1 to 3 μm within a 15-min period, compared to particle vibrations, with a net displacement of <1 μm (Fig. 4B) and α = 1.59 (see Fig. S1 in the supplemental material).

**TABLE 5:** Viscoelasticity and creep compliance of 19-h biofilms formed by *P. aeruginosa* and matrix polysaccharide mutants with *S. aureus*, respectively

Biofilm	Region	α	*J*(*t*) (*t* = 101 s, Pa^−1^)
Medium (negative control)	Liquid phase	1.05	11,500 ± 789
wtPAO1-*S. aureus*	Microcolony	0.87	3 ± 2
Mutant Δ*pelA*-*S. aureus*	Microcolony	0.74	2 ± 0
Mutant Δ*pslBCD*-*S. aureus*	Loose microcolony	0.41	1,294 ± 109

10.1128/mBio.00585-18.2 FIG S1 MSD curve for particles undergoing superdiffusion in wild-type P. aeruginosa-S. aureus microcolonies. The orange dotted line indicates the line of best fit to the experimentally determined MSD curve determined by the use of a power law function for the estimation of α. Error bars represent standard errors of the means (SEM). Download FIG S1, PDF file, 0.2 MB. Copyright © 2018 Chew et al. 2018 Chew et al. This content is distributed under the terms of the Creative Commons Attribution 4.0 International license.

The Δ*pelA*-S. aureus microcolonies had lower particle MSDs and thus were more effectively cross-linked than wild-type P. aeruginosa-S. aureus biofilm microcolonies (Fig. 4A). None of the particles were observed to undergo superdiffusion (Fig. 4B). The microcolonies had a creep compliance *J *=* *2 ± 0 Pa^−1^ at *t *=* *10^1^ s and were more elastic (α = 0.74) (Table 5).

Particles in mutant Δ*pslBCD*-S. aureus microcolonies were less confined, indicating a reduction in cross-linking (Fig. 4B). Mutant Δ*pslBCD*-S. aureus biofilms had creep compliance of 1,294 ± 109 Pa^−1^ and were more elastic (α = 0.41) (Table 5). The viscoelastic properties of the microcolonies formed in the dual-species P. aeruginosa-S. aureus and mutant Δ*pelA*-S. aureus biofilms in this study were different from those seen with the monospecies P. aeruginosa biofilms cultivated under flow conditions, as previously reported (33), where the microcolonies were elastic. However, the cross-linking role of Psl and matrix loosening mediated by Pel in monospecies biofilms were maintained in the P. aeruginosa-S. aureus community, regardless of flow conditions. The reduced matrix stiffness conferred by Pel may have contributed to the superdiffusive microenvironment for enabling microcolony expansion and increased surface coverage.

### The competitive fitness of P. aeruginosa requires DGC SiaD.

According to the selection constant rates (*r_ij_*), changes in the extent of competitiveness of the Δ*pelA* mutant over S. aureus were similar to those seen with wild-type P. aeruginosa during dual-species biofilm development, whereas Δ*pslBCD* and Δ*pelA* Δ*pslBCD* mutants were less competitive in early biofilms (Fig. 2B) (Table 3, columns 5 and 6). This suggests that Psl is important for the competitive fitness of P. aeruginosa during early biofilm development. Psl has an active signaling role in biofilm formation, where it stimulates the activity of two DGCs, SiaD and SadC, thereby elevating intracellular c-di-GMP content (31, 32). This mechanism may also facilitate the competitiveness of P. aeruginosa in the P. aeruginosa-S. aureus community (31, 32). Indeed, Δ*pslBCD* mutants are delayed in biofilm development (25), which may have allowed S. aureus to form microcolonies in the dual-species biofilm.

**FIG 5:**
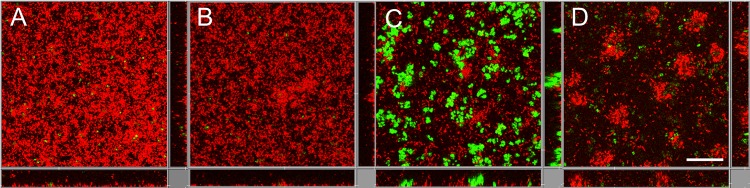
Confocal images of 19-h biofilms of P. aeruginosa CdrA and diguanylate cyclase mutants cocultured with S. aureus. (A) P. aeruginosa Δ*cdrA*-S. aureus. (B) P. aeruginosa Δ*sadC*-S. aureus. (C) P. aeruginosa Δ*siaD*-S. aureus. (D) P. aeruginosa Δ*siaD*(*siaD*)-S. aureus. P. aeruginosa was mCherry tagged (red), and S. aureus was Gfp tagged (green). Images are representative of results from at least three biological replicates. The scale bar is 30 μm.

**FIG 6:**
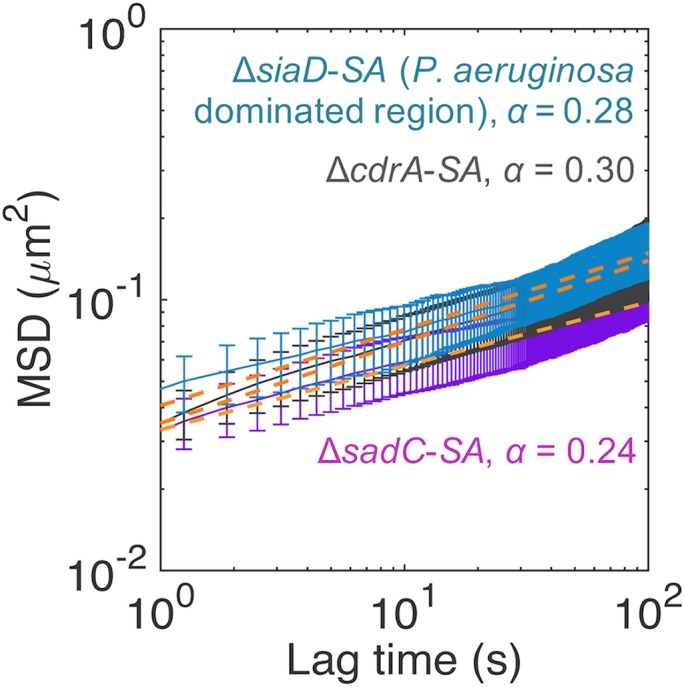
MSD curves for P. aeruginosa Δ*siaD*-S. aureus, P. aeruginosa Δ*cdrA*-S. aureus, and P. aeruginosa Δ*sadC*-S. aureus microcolonies. *SA*, S. aureus. The orange dotted lines indicate the line of best fit to the experimentally determined MSD using a power law function for the estimation of α. Error bars represent SEM.

P. aeruginosa competitiveness may also be associated with other molecules that interact with Psl. CdrA is a P. aeruginosa adhesin that binds to Psl and cross-links Psl polysaccharide polymers to increase the structural stability of the biofilm (28). We thus examined the effects of SiaD and SadC as well as of CdrA on P. aeruginosa competitiveness. The Δ*cdrA* and Δ*sadC* mutants dominated the dual-species biofilms, indicating that CdrA and SadC were not essential for P. aeruginosa competitiveness (Fig. 5A and B). Their fitness curves are given in Fig. S2. The biofilms were largely flat and undifferentiated, with small microcolonies (Fig. 5B). The loss of the SiaD DGC resulted in a significant reduction in competitive fitness for P. aeruginosa in mutant Δ*siaD*-S. aureus biofilms, with *r_ij_* = 0.07 ± 0.04 at 12 h and *r_ij_* = 0.01 ± 0.01 at 19 h, which were different from the results seen with the wild-type strain (*P* < 0.01, α = 0.05, *n* = 4) (Table 3, columns 2 to 4) (Fig. S2). There was little colocalization of P. aeruginosa and S. aureus in the biofilms, and S. aureus formed homogenous microcolonies devoid of P. aeruginosa Δ*siaD* cells (Fig. 5C). The rheological properties of these microcolonies, with monospecies S. aureus microcolonies investigated for comparison, are shown in Table S1 and Fig. S3 in the supplemental material. Genetic complementation of the Δ*siaD* mutant restored P. aeruginosa competitiveness (*r_ij_* = 0.18 ± 0.05), and the formation of microcolonies was dominated by P. aeruginosa after 19 h (Table 3, columns 2 to 4) (Fig. 5D). P. aeruginosa dominated regions of mutant Δ*cdrA-*S. aureus, mutant Δ*sadC-*S. aureus and mutant Δ*siaD-*S. aureus biofilms (Fig. 5) and had higher particle MSDs than the wild-type strain but less than the mutant Δ*pslBCD*-S. aureus microcolonies (Fig. 6). Thus, Psl remained the major contributor of matrix cross-linking although CdrA and c-di-GMP, though the SadC and SiaD signaling pathway also contributed to matrix cross-linking and biofilm stability. Further details of the number and size of microcolonies and of the rheological properties of the biofilm structures are given in Table S1.

10.1128/mBio.00585-18.3 FIG S2 Fitness of P. aeruginosa mutants Δ*cdrA*, Δ*sadC*, and mutant Δ*siaD* and complemented strain Δ*siaD*(*siaD*) relative to S. aureus. A selection constant of *r_*ij*_* = 0 means that P. aeruginosa and S. aureus are equally competitive and that of *r_*ij*_* > 0 means P. aeruginosa is more competitive than S. aureus. Asterisks (*) indicate a significant difference. Download FIG S2, PDF file, 0.01 MB. Copyright © 2018 Chew et al. 2018 Chew et al. This content is distributed under the terms of the Creative Commons Attribution 4.0 International license.

10.1128/mBio.00585-18.4 FIG S3 MSD curves for S. aureus microcolonies in mutant Δ*siaD*-S. aureus dual-species biofilms compared to monospecies S. aureus microcolonies*. SA*, S. aureus. The orange dotted lines indicate the line of best fit to the experimentally determined MSD curve determined by the use of a power law function for the estimation of α. Error bars represent SEM. Download FIG S3, PDF file, 0.1 MB. Copyright © 2018 Chew et al. 2018 Chew et al. This content is distributed under the terms of the Creative Commons Attribution 4.0 International license.

10.1128/mBio.00585-18.5 TABLE S1 Microcolony formation and microrheological properties of 19-h biofilms formed by mutant Δ*cdrA* and diguanylate cyclase mutants of P. aeruginosa with S. aureus. Download Table S1, DOCX file, 0.01 MB. Copyright © 2018 Chew et al. 2018 Chew et al. This content is distributed under the terms of the Creative Commons Attribution 4.0 International license.

### SiaD-mediated competition is siderophore and PQS independent.

**FIG 7:**
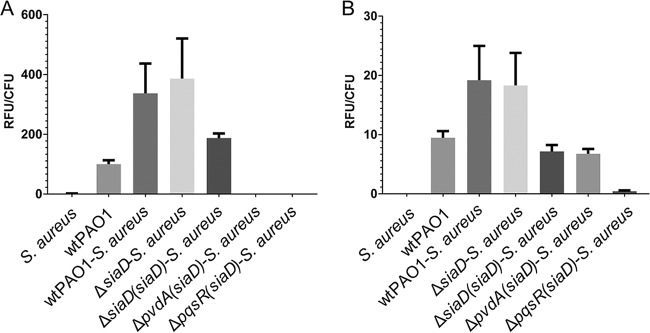
Relative levels of pyoverdine and PQS in 19-h P. aeruginosa-S. aureus biofilms. (A) Pyoverdine, as indicated by its fluorescence at an emission peak of 450 nm. (B). PQS, as indicated by the fluorescence emission of the green fluorescent protein from the PQS biosensor strain, mutant Δ*pqsC*(*pqsA*-*gfp*). Values are means (± standard deviations [SD]) of relative fluorescence units (RFU) determined from three biological replicates.

P. aeruginosa uses the iron siderophore pyoverdine and products of *pqs* genes, such as N-oxo-2-heptyl-4-hydroxyquinoline (HQNO), to outcompete S. aureus in cocultures (18, 43). Hence, we examined whether the P. aeruginosa SiaD-mediated competition with S. aureus involved pyoverdine and PQS expression. The pyoverdine levels of the dual-species biofilms were estimated by measuring fluorescence at 450 nm, the peak level of emission for pyoverdine (44). The PQS levels were determined based on Gfp fluorescence using a PQS biosensor strain, Δ*pqsC*(*pqsA*-*gfp*), where the Δ*pqsC* mutant is unable to synthesize its own PQS and *pqsA*-*gfp* can only be induced by exogenously added PQS (45). Using these approaches, we found that the levels of production of pyoverdine (Fig. 7A) and PQS (Fig. 7B) of wild-type P. aeruginosa and the SiaD mutant were similar and that complementation of SiaD resulted in pyoverdine and PQS levels lower than those measured for the wild type. These results suggest that the inability of the SiaD mutant to compete with S. aureus was not due to a deficiency of pyoverdine and PQS production.

## DISCUSSION

Coinfections of P. aeruginosa and S. aureus are often found in patients with cystic fibrosis (CF) and with diabetic and chronic wounds. Such mixed-species infections are correlated with poor clinical outcomes (16); hence, these two organisms serve as a model, dual-species community to represent polymicrobial infections (18, 43, 46). The interactions between the two organisms affect the efficacy of antimicrobial treatments (46). P. aeruginosa has been found to compete with S. aureus for oxygen and to induce the bacterium to shift to fermentative metabolism to produce lactate, which P. aeruginosa consumes (18). P. aeruginosa can also lyse S. aureus to obtain iron. This is mediated through induction of PQS-dependent virulence genes as well as the production of siderophores (43). However, detailed investigation of interspecies interactions during dual-species biofilm formation and the role of the biofilm matrix in enabling interspecies interactions and determining community structure have been less extensively explored.

In this study, P. aeruginosa restricted S. aureus growth and biofilm formation, whereas in the presence of S. aureus, P. aeruginosa showed an increased surface coverage and number of microcolonies. Further, P. aeruginosa microcolonies were larger and the overall biovolume was higher when S. aureus was present. Similar observations were previously reported for these dual-species combinations, where it was suggested that P. aeruginosa lysed S. aureus to be used as a nutrient source (18, 43). Pel and Psl polysaccharides were not required for P. aeruginosa to outcompete S. aureus, which was similar to the result seen with mucoid P. aeruginosa*-*S. aureus biofilms (33). Nevertheless, distinct effects contributed by the polysaccharides, such as reduced formation of S. aureus microcolonies during early biofilm development (mediated by Psl) and increased biofilm biovolumes of P. aeruginosa during mid- to late-stage biofilm development by Pel, were observed. These findings are consistent with a shift in production with time of biofilm formation, from Psl to Pel, similar to that documented for monospecies biofilms (33). In addition, the data indicate that Pel increases surface coverage throughout biofilm development and expands the microcolony size in mature biofilms. This finding aligns with that of our previous study, i.e., that Pel enhances spreading in monospecies biofilms (33). The latter study also attributed the differences in biofilm structures to Psl generating a more elastic and cross-linked matrix and Pel contributing a loose and viscoelastic matrix. Indeed, in agreement with the results obtained for monospecies biofilms, we found that Psl increased the effective cross-linking of the microcolonies in dual-species biofilms (Fig. 4), making them less compliant (Table 5) and more compact (Fig. 1). Psl was the major contributor of effective cross-linking in the biofilm, while the CdrA adhesin and biofilm regulatory components SadC and SiaD played less of a role (Fig. 6) (see also Table S1 in the supplemental material).

Interestingly, the microcolonies that expressed only Pel were more elastic than the microcolonies that expressed only Psl, and the microcolonies that expressed both Pel and Psl were the most viscous. This indicated that the rheological contributions of the polysaccharides were not simply additive and that the physical structure changed in the absence of one of these polysaccharides. In addition to changes in the mechanical properties of the biofilm in the presence of S. aureus, it is possible that the Psl matrix is more viscous when it is produced under static versus flow conditions. This may reflect observations of biofilms on rocks beneath waterfalls that are constantly exposed to high shear (47, 48). The expression levels of both Pel and Psl were associated with superdiffusion of particles and with a more compliant biofilm matrix (Fig. 4) (Table 5). The superdiffusion of particles could be the result of fast movement of cells. P. aeruginosa is known to be motile, mediated by swimming, swarming, and twitching based processes. Superdiffusion could also be the result of particles travelling directionally through biofilm channels. For example, Birjiniuk et al. (2014) observed particle trajectories that indicated that the particles were travelling from top to the bottom of the biofilm through interconnected fluid-filled microscale channels (49).

The ability of Psl to initiate P. aeruginosa biofilm formation and mediate competitive fitness may be linked to its ability to facilitate biofilm formation through activating DGCs, leading to c-di-GMP production. Indeed, the SiaD DGC, activated by Psl (31), was critical for P. aeruginosa competitiveness in the dual-species biofilms (Fig. 5). Without SiaD, P. aeruginosa and S. aureus were equally competitive, with *r_ij_* = 0.01 ± 0.04 at 19 h, with S. aureus establishing many microcolonies in the dual-species biofilm (Fig. 5). Moreover, SiaD induces autoaggregation in P. aeruginosa when exposed to SDS stress (50) and /tellurite (TeO_3_^2–^) (32). In a previous study (50), Psl was found to be essential for autoaggregation. Hence, it is possible that SiaD is activated by exoproducts from S. aureus, providing a mechanism by which P. aeruginosa can sense S. aureus to induce autoaggregation and biofilm formation.

P. aeruginosa is known to outcompete S. aureus using the siderophore pyoverdine and downstream products of the PQS biosynthetic pathway in planktonic cultures (18, 43). In monospecies P. aeruginosa biofilms, SiaD has been found to negatively control pyoverdine production (51). Similarly, high c-di-GMP concentrations reduce PQS production (52). Thus, it was unexpected that the production levels of pyoverdine and PQS in the Δ*siaD* mutant were not increased but rather were similar to those seen with wild-type P. aeruginosa biofilm cocultures (Fig. 7). Complementation of the *siaD* mutant resulted in pyoverdine and PQS levels similar to the levels seen with wild-type P. aeruginosa monospecies biofilms but not dual-species wild-type biofilms (Fig. 7). This indicated that the overproduction of PQS in the *siaD* mutant does not play a significant role in the competitive phenotype here and that the impact of overproducing SiaD and, hence, of elevated c-di-GMP levels drives competition through another factor that is siderophore and PQS independent. Further investigation is required to understand the underlying mechanism of how SiaD activity increases the competitiveness of P. aeruginosa.

**FIG 8:**
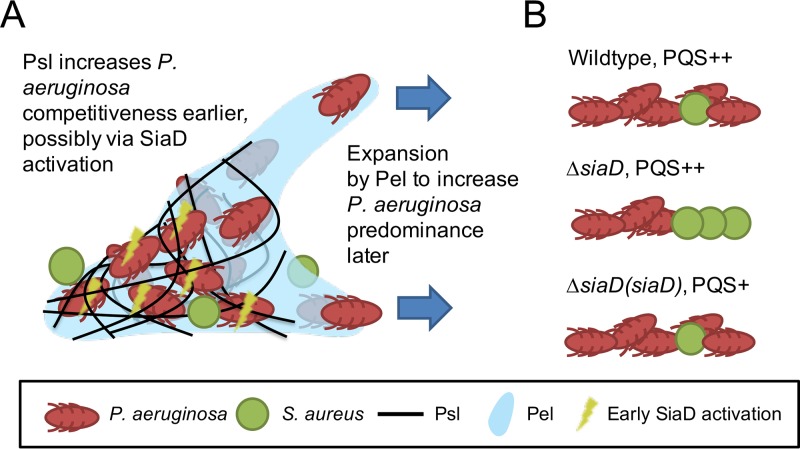
Schematic showing how matrix polysaccharides and SiaD contribute to P. aeruginosa predominance in dual-species P. aeruginosa-S. aureus communities. (A) Psl enhances P. aeruginosa competitiveness in early stages, possibly via SiaD activation, whereas Pel enables biofim expansion to increase P. aeruginosa predominance in the later stages. (B) Dominance of wild-type P. aeruginosa and SiaD and SiaD complement mutant over S. aureus, with their corresponding PQS/pyoverdine (PVD) levels.

The findings presented here provide novel information on the mechanisms by which the P. aeruginosa-S. aureus dual-species biofilms are established and how P. aeruginosa dominates the community during biofilm development. We summarize the findings in Fig. 8, where we show that the Psl polysaccharide is required for initial competition whereas the Pel polysaccharide enables the predominance of P. aeruginosa in mature, dual-species biofilms (Fig. 8A). It is also clear that the SiaD cyclase is important for P. aeruginosa competitiveness, which occurs in a pyoverdine- and PQS-independent fashion, with the *siaD* mutant producing amounts similar to those produced by the wild-type strain and the complemented *siaD* mutant producing less than the wild-type, dual-species biofilms (Fig. 8B). This highlights the fact that the regulatory mechanisms governing competition between P. aeruginosa and S. aureus are likely to be complex, incorporating recognition of a competitor and temporal regulation of different factors that impact the dual-species interactions. These results help to increase understanding of the mechanisms by which these two opportunistic pathogens interact during biofilm formation and could suggest strategies for the control of dual-species infections.

## MATERIALS AND METHODS

### Bacterial strains.

The bacterial strains and plasmids used in this study are listed in Table 1. Overnight cultures of P. aeruginosa were grown in 100% LB medium (10 g liter^−1^ NaCl, 5 g liter^−1^ yeast extract, and 10 g liter^−1^ tryptone) at 37°C with shaking (200 rpm). S. aureus was grown in 100% TSB medium (17 g liter^−1^ casein peptone, 2.5 g liter^−1^ K_2_HPO_4_, 2.5 g liter^−1^ glucose, 5 g liter^−1^ NaCl, 3 g liter^−1^ soya peptone) at 37°C with shaking (200 rpm).

### Construction of Pseudomonas aeruginosa mutants.

The Δ*sadC* and Δ*siaD* mutants, defective in production of SadC and SiaD diguanylate cyclases, respectively, were constructed by homologous recombination as previously described (51, 53). The Δ*cdrA* mutant, defective for the CdrA adhesin, was constructed by homologous recombination using lambda Red recombinase as previously described (54) with a Multisite Gateway (Thermo Fisher Scientific, MA) LR expression clone containing the PCR product 5′ CdrA upstream fragment, gentamicin gene, 3′ CdrA downstream fragment (primers 5′ CdrA upstream F [GGGG ACA ACT TTG TAT AGA AAA GTT G AGGGTCTTGCCTTCCAGTTC] and R [GGGG AC TGC TTT TTT GTA CAA ACT TG GAAAATCTCCCTATCTGCGTGG] and 3′ CdrA downstream F [GGGG ACA GCT TTC TTG TAC AAA GTG G TCCTCGAAAACCCGTTCCTG] and R [GGGG AC AAC TTT GTA TAA TAA AGT TG CTTCGTATCGCTGCTGTTGC]). A pUCP18-*siaD* plasmid (51) was used to genetically complement the P. aeruginosa Δ*siaD* mutant.

### Cultivation of static biofilms.

Overnight cultures of P. aeruginosa and S. aureus were diluted with TSB medium to optical densities at 600 nm (OD_600_) of 0.01 and 0.02, respectively, to yield cell densities of approximately 2 × 10^7^ CFU ml^−1^. For monospecies biofilm cultivation, µ-Side eight-well microscopy chambers (ibidi, Martinsried, Germany) were inoculated with 200 µl of diluted overnight cultures of P. aeruginosa or S. aureus. For dual-species biofilms, µ-Slide eight-well microscopy chambers were inoculated with 100 µl of P. aeruginosa and S. aureus each to give total initial cell densities similar to those of the monospecies biofilms with 1:1 ratios. The cultures were incubated at 37°C under static conditions.

### Biofilm image acquisition and analysis.

Biofilms were visualized using a Zeiss LSM780 confocal scanning laser microscopy (Oberkochen). P. aeruginosa strains were fluorescently marked using miniTn7-mCherry (55). mCherry was detected using an argon laser for excitation at a wavelength of 568 nm and a low-pass emission filter at a wavelength of 590 nm. S. aureus 15981 was fluorescently marked using pSB2019, expressing Gfp (56). Gfp was detected using an argon laser for excitation at a wavelength of 488 nm and a broad-pass emission filter at wavelengths of 500 to 530 nm. Images were reconstructed using the Imaris software package (Bitplane, AG), and the biovolumes, microcolony numbers and sizes, and surface coverage values were calculated using COMSTAT (www.comstat.dk; see Table S1 in the supplemental material) (57, 58). Biovolumes were measured and calculated from four biological replicates, whereas microcolony sizes and surface coverages were calculated from three biological replicates. Each biological replicate was derived from an average of three confocal images. The mean and variance of microcolony sizes were derived after logarithmic transformation of the data according to their lognormal distribution (59). Significant differences between the distributions of microcolony sizes were determined by the Kolmogorov-Smirnov test. The competitiveness of bacterial species *i* over *j* was expressed as the selection rate constant (*r_ij_*), which was calculated according to the equation $r_{ij}=\frac{ln\left(\frac{N_{i}(t)}{N_{i}(0)}\right)-\text{ln}\left(\frac{N_{j}(t)}{N_{j}(0)}\right)}{t}$, where *t* is time in hours and *N* is the biovolume of species *i* or *j* at the start (*t *=* *0) or at time *t* (60). The competitiveness levels of the two different species are equal when *r_ij_* = 0.

### Microrheology.

Fluorescent latex beads 1.0 µm in diameter and with carboxylate modification (Invitrogen, CA) were dispersed in TSB medium to reach a final concentrations of 18.2 × 10^6^ particles ml^−1^, and medium was used to dilute the overnight cultures and to grow biofilms. After particle incorporation into the biofilms, their movement was tracked by fluorescence microscopy with a 63× objective (Zeiss Axio Observer Z1). The motion of particles in the cocultures was captured in 15-min videos at frame rates of 1 to 5 fps. The particle trajectories were obtained with ImageJ (https://fiji.sc/) (61) plugin Mosaic Particle Tracker (62). The trajectories were pooled for each biofilm, and the mean squared displacement (MSD) values were calculated and analyzed using msdanalyzer (63), a MATLAB class for MSD analysis. The MSD is proportional to the creep compliance, *J*(*t*), of the material according to the following relation:

$$J\left(t\right)=\frac{3\pi d}{4k_{B}T}\text{MSD}\left(t\right)$$

where *J* = creep compliance, *d* = particle diameter, *k_B_* = Boltzman constant, and *T* = temperature (37, 39). The microrheological properties are related to the MSD levels, which in our work here are well described by MSD (*t*) ∼ *t*^α^ (37, 38, 41). The values of α were extracted from the logarithmic fit to the MSD for a lag time range of approximately 1 s to 100 s. The *R*^2^ values of the curves were greater than 0.96.

### Pyoverdine and Pseudomonas quinolone signal (PQS) assay.

Monospecies and dual-species biofilms were grown in 24-well plates for 19 h (Nunc 142475 Nunclon). The wells were centrifuged at 13,000 × *g* for 3 min to separate and obtain cell pellets and supernatants. The cell pellets were resuspended and plated onto Pseudomonas isolation agar for CFU counting of P. aeruginosa cells. Cells from monospecies S. aureus biofilms were plated on TSB agar for CFU counting. The supernatants filtered with 0.2-µm-pore-size Acrodisc PF syringe filters with Supor membrane (Pall Life Sciences, USA). For the pyoverdine assay, the relative levels of pyoverdine in the supernatants were estimated from their emission fluorescence at 450 nm using laser excitation at 400 nm with a Magellan Tecan Infinite 200 Pro microplate reader (Männedorf). For the PQS assay, 100 µl of 50× diluted overnight cultures of PQS biosensor strain Δ*pqsC*(*pqsA*-*gfp*) was mixed with 100 µl of the filtered biofilm supernatants. The mixtures were then cultivated in a 96-well microplate at 37°C. Green fluorescent protein (GFP) fluorescence from *pqsA*-*gfp* expression was measured using a Magellan Tecan Infinite 200 Pro microplate reader (Männedorf) to indicate PQS levels. All emission fluorescence readings were normalized to the CFU of P. aeruginosa.

10.1128/mBio.00585-18.1 TEXT S1 Supplemental Methods. Download Text S1, DOCX file, 0.01 MB. Copyright © 2018 Chew et al. 2018 Chew et al. This content is distributed under the terms of the Creative Commons Attribution 4.0 International license.
