# Supplementary figures and images for: Matrix Polysaccharides and SiaD Diguanylate Cyclase Alter Community Structure and Competitiveness of Pseudomonas aeruginosa during Dual-Species Biofilm Development with Staphylococcus aureus
Source: mBio. 2018 Nov 6;9(6):e00585-18. doi: 10.1128/mBio.00585-18 (PMC6222129; doi:10.1128/mBio.00585-18)

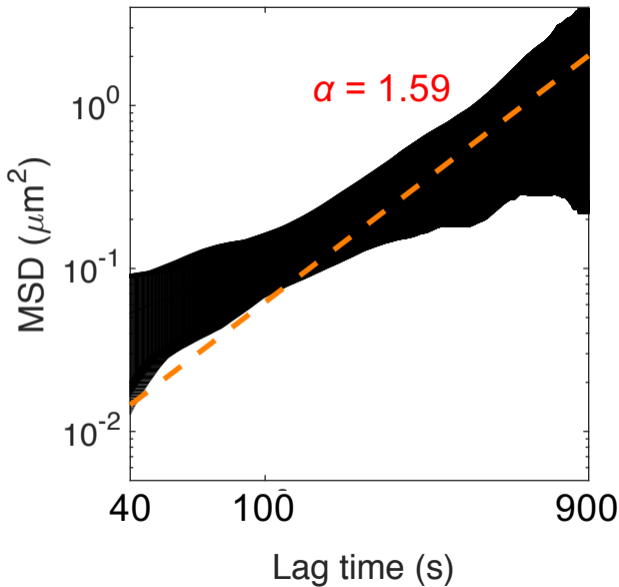

Supplement: FIG S1 [file mbo005184153sf1.pdf]

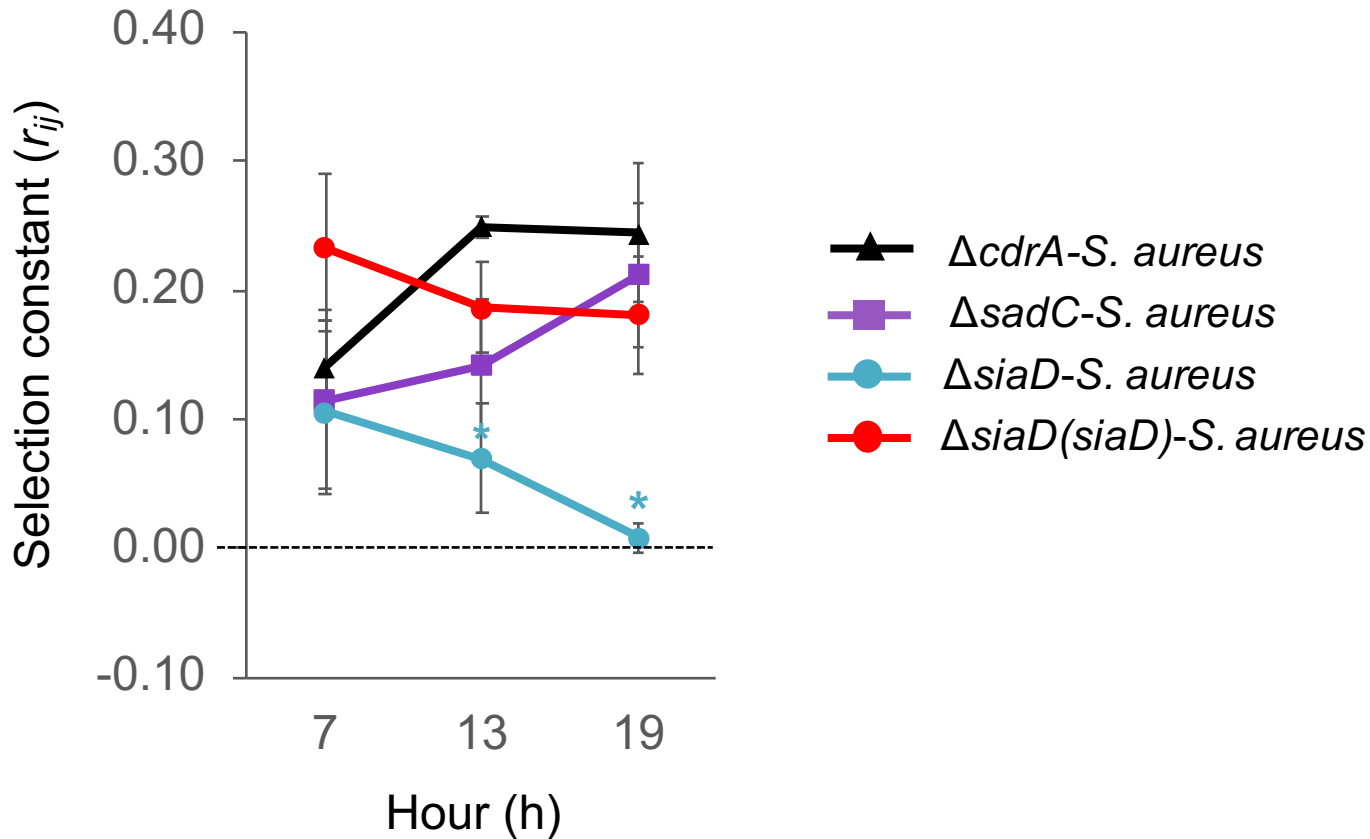

Supplement: FIG S2 [file mbo005184153sf2.pdf]

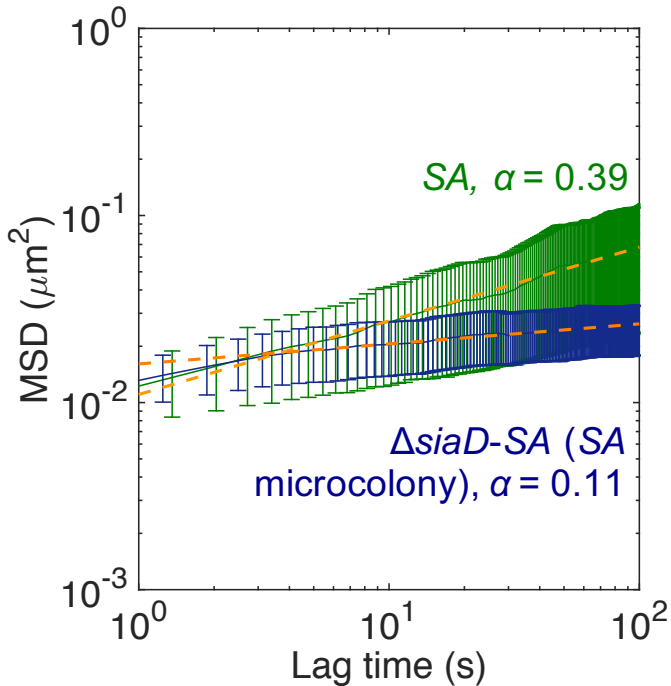

Supplement: FIG S3 [file mbo005184153sf3.pdf]
