# Supplementary material for: Matrix Polysaccharides and SiaD Diguanylate Cyclase Alter Community Structure and Competitiveness of Pseudomonas aeruginosa during Dual-Species Biofilm Development with Staphylococcus aureus
Source: mBio. 2018 Nov 6;9(6):e00585-18. doi: 10.1128/mBio.00585-18 (PMC6222129; doi:10.1128/mBio.00585-18)
Supplement: TABLE S1 [file mbo005184153st1.docx]

**Supplementary Tables**

Table S1. Microcolony formation and microrheological properties of 19 h biofilms formed by *cdrA-* and diguanylate cyclase mutants of *P. aeruginosa* with *S. aureus*.

|  | Microcolony Formation Properties | | Microrheological Properties | |
| --- | --- | --- | --- | --- |
| Biofilm | Average No. of Microcolony per Area^†^ (mm^-2^) | Average Microcolony Biovolume^‡^ (μm^3^) | α | *J(t*)  (*t* = 10^1^ s, Pa^-1^) |
| Δ*cdrA*-*S. aureus* | 5,528 ± 2,265 | 140 ± 16 | 0.30 | 41 ± 9 |
| Δ*sadC*-*S. aureus* | 4,420 ± 1,318 | 117 ± 20 | 0.24 | 34 ± 8 |
| Δ*siaD*-*S. aureus* (with respect to *S. aureus* microcolony) | 4,539 ± 350 | 325 ± 23 | 0.11 | 12 ± 4 |
| Δ*siaD*-*S. aureus* (with respect to *P. aeruginosa* dominated region and microcolony) | 3,020 ± 1,972 | 130 ± 8 | 0.28 | 44 ± 11 |
| Monospecies *S. aureus* | 2,642 ± 178 | 7,356 ± 2279 | 0.39 | 16 ± 8 |
