# Supplementary material for: Matrix Polysaccharides and SiaD Diguanylate Cyclase Alter Community Structure and Competitiveness of Pseudomonas aeruginosa during Dual-Species Biofilm Development with Staphylococcus aureus
Source: mBio. 2018 Nov 6;9(6):e00585-18. doi: 10.1128/mBio.00585-18 (PMC6222129; doi:10.1128/mBio.00585-18)
Supplement: TEXT S1 [file mbo005184153s1.docx]

**Supplementary Methods**

**Calculation of microcolony sizes in COMSTAT**

Microcolonies had a minimum threshold base area of 100 pixels or 7 μm^2^ in order for COMSTAT to calculate their biovolumes. The minimum threshold base area was unchanged from the initial COMSTAT setting and roughly corresponds to the base area of a hemisphere with a biovolume of 10 *P. aeruginosa* cells with an average length and radius of 2.25 μm and 0.325 μm, respectively.
